# Influence of parental resilience on non-suicidal self-injury in adolescent cancer patients

**DOI:** 10.3389/fpsyt.2025.1618280

**Published:** 2025-09-10

**Authors:** Xiang Huang, Huiyun Li, Miaomiao Wu, Zheng Chen, Yufei Sang

**Affiliations:** ^1^ Nursing Department, 923 Hospital of Joint Logistic Support Force of PLA, Nanning, China; ^2^ Department of Disease Control and Prevention, 923 Hospital of Joint Logistic Support Force of People's Liberation Army (PLA), Nanning, China; ^3^ Medical service Department, 923 Hospital of Joint Logistic Support Force of PLA, Nanning, China

**Keywords:** resilience, adolescents, non-suicidal self-injury, scale, influence

## Abstract

**Objective:**

To examine the effect of a structured, DBT-based parental resilience training program on non-suicidal self-injury (NSSI) and related psychological symptoms in adolescents with cancer.

**Methods:**

This pre–post study without a control group enrolled 38 adolescent cancer patients (aged 13–18) with a history of NSSI, along with one of their parents. From March 2023 to May 2024, parents participated in a 4-week intervention consisting of three structured skills-training sessions (90 minutes each), weekly family group skills training, ongoing telephone counseling, and weekly consultations with a psychological support team. The Ottawa Self-Injury Scale was used to assess NSSI recurrence in adolescents. Anxiety and depression symptoms in adolescents were measured using the Generalized Anxiety Disorder Scale (GAD-7) and the Patient Health Questionnaire (PHQ-9). Parental resilience was evaluated with the Connor-Davidson Resilience Scale (CD-RISC).

**Results:**

All administered questionnaires were fully completed and valid, yielding a 100% response rate. Following the 4-week intervention, the recurrence rate of NSSI was 13.16% (5/38). Adolescents demonstrated significant reductions in both GAD-7 and PHQ-9 scores from pre- to post-treatment (P < 0.05), indicating marked improvements in anxiety and depression symptoms. Parental CD-RISC scores increased significantly (P < 0.05), reflecting enhanced resilience. Subgroup analyses revealed that these improvements were significant across both genders and age groups (<15 years vs. ≥15 years), with no statistically significant differences in the magnitude of change between subgroups (P > 0.05).

**Conclusions:**

A short-term, DBT-based parental resilience training program may improve caregiver resilience, reduce adolescent anxiety and depression, and lower short-term NSSI recurrence in adolescents with cancer. However, the lack of a control group and short follow-up limit the generalizability of findings. Further controlled studies with larger samples and longer follow-up are warranted.

## 1 Introduction

Non-suicidal self-injury (NSSI) refers to deliberate, direct harm to one’s own body tissues without suicidal intent, including behaviors such as cutting, burning, scratching, or hitting oneself (1). Although not intended to cause death, NSSI is strongly associated with emotional distress, functional impairment, and increased suicide risk. Adolescence is a developmental stage marked by significant emotional, psychological, and social changes, during which individuals are particularly vulnerable to mental health challenges such as depression, anxiety, peer pressure, and identity struggles. Consequently, NSSI frequently emerges during this period, with prevalence rates consistently higher among adolescents than in adults (2).

Multiple factors contribute to the onset and persistence of NSSI in adolescents, including mental health disorders such as depression, anxiety, and borderline personality disorder; trauma and abuse, with histories of physical, emotional, or sexual abuse significantly elevating risk; peer pressure and social media, where certain online communities may normalize or encourage self-injurious behaviors; and family dynamics, as dysfunctional environments with poor communication and limited emotional support can heighten vulnerability (3).

Among these, family context is especially significant, as it represents the primary environment in which adolescents develop. Emerging evidence suggests that parental psychological resilience—the capacity to adapt positively to stress—can buffer adolescents from engaging in NSSI. Parental resilience has been linked to better emotional regulation in adolescents, reduced suicide attempts, and improved intervention outcomes (4, 5). Strengthening resilience in parents may therefore offer a promising avenue for family-based interventions.

However, while existing literature extensively addresses NSSI in general adolescent populations, few studies have focused on adolescents facing chronic, life-threatening illnesses such as cancer. Adolescents with cancer are exposed to unique stressors—prolonged hospitalization, treatment side effects, social isolation, and existential distress—that may amplify emotional vulnerability and risk for NSSI. Family resilience may be especially critical in this context, yet research examining its protective role for adolescent cancer patients remains scarce. This gap limits the development of targeted, evidence-based interventions for this high-risk group.

This research is grounded in the theoretical framework of DBT and psychological resilience theory. DBT, developed by Linehan, is rooted in cognitive-behavioral principles and emphasizes the dialectical process of balancing acceptance with change. Its core components—emotional regulation, distress tolerance, interpersonal effectiveness, and mindfulness—are particularly relevant for adolescents engaging in NSSI, as these skills directly address the maladaptive coping patterns and intense affective states associated with self-injurious behavior. Psychological resilience theory complements DBT by focusing on the dynamic capacity of individuals to adapt positively in the face of adversity. Resilience is not a fixed trait but a process influenced by individual, familial, and social factors, and can be enhanced through targeted interventions. By integrating DBT’s skill-based strategies with resilience-building for parents, this study is based on the premise that strengthening the emotional and coping capacities of caregivers can indirectly improve adolescents’ mental health and reduce the recurrence of NSSI. This dual focus aligns with ecological and family systems perspectives, which emphasize that adolescent behavior is shaped not only by individual characteristics but also by the surrounding family environment.

The present study aims to investigate the relationship between parental resilience and NSSI among adolescent cancer patients, with a focus on determining the prevalence of NSSI in this population, examining how parental resilience is associated with NSSI occurrence, and exploring whether family environment factors mediate or moderate this relationship. Specifically, we hypothesize that (H1) higher levels of parental resilience will be associated with a lower prevalence of NSSI in adolescent cancer patients, and (H2) positive family environment factors will mediate the relationship between parental resilience and reduced NSSI. The findings may help identify family-based strategies to mitigate self-injurious behaviors and improve mental health outcomes in this vulnerable population.

## 2 Materials and methods

### 2.1 Participants

Adolescent patients (aged 13–18 years) presenting with non-suicidal self-injury (NSSI) at psychiatric outpatient clinics between March 2023 and May 2024 were recruited if they met the following criteria:

1. Documented NSSI behaviors without suicidal intent;
2. Current prescription of psychotropic medication(s);
3. ≤3 prior sessions of individual psychotherapy;
4. Availability of ≥1 parent committed to completing resilience training.

### 2.2 Exclusion criteria

#### 2.2.1 Patients were excluded if they exhibited

1. Diagnoses of severe psychiatric disorders (e.g., schizophrenia, bipolar I disorder);
2. Suicide attempts within 30 days preceding enrollment;
3. Parental withdrawal from training participation;
4. Discontinuation of clinical follow-up during the study period.

A total of 38 adolescent patients (9 males, 29 females; mean age 15.63 ± 2.58 years) met eligibility requirements. For each adolescent, one parent participated (mother: n=27, father: n=11). The mean age of participating parents was 42.1 ± 5.8 years. Demographic information about the participating parents — including age, educational attainment, occupation, and primary caregiving role — was collected and is presented in **Table 1**. The sample screening procedure is outlined in **Table 2**. Informed consent and assent were obtained, with both the adolescents (via assent) and their parents or guardians (via consent) signing forms to confirm their understanding and willingness to participate in the study.

**Table 1:** Parent demographic characteristics (n = 38).

Characteristic	Category	n (%) or Mean ± SD
Age (years)	Mean ± SD	42.1 ± 5.8
Education Level	High school or below	12 (31.6)
	College	20 (52.6)
	Postgraduate	6 (15.8)
Occupation	Employed	24 (63.2)
	Unemployed	8 (21.1)
	Other	6 (15.8)
Primary Caregiving Role	Mother	27 (71.1)
	Father	11 (28.9)

**Table 2:** Sample screening workflow.

Step	Activity	Responsible party
1	Identify potential participants (clinician referrals, self-report, clinic database search)	Clinician or Research Team
2	Initial contact and invitation to participate	Research Team
3	Informed consent and assent (adolescent and parent)	Research Team
4	Eligibility screening (interviews, review of medical records)	Clinician or Research Team
5	Confirm inclusion and exclusion criteria	Research Team
6	Parent and adolescent orientation	Research Team
7	Final enrollment and baseline data collection	Research Team

### 2.3 Intervention methods

The parental intervention was based on Dialectical Behavior Therapy (DBT) principles (6), with a focus on resilience enhancement. DBT’s dialectical approach—balancing acceptance and change—targets emotional regulation, interpersonal effectiveness, and distress tolerance. Sessions were conducted in person in the clinic’s family therapy room; for parents unable to attend physically, synchronous online video sessions via an encrypted platform ensured continuity. The program was delivered by two licensed clinical psychologists with specialized training in DBT and family therapy, supported by a psychiatric nurse responsible for coordination and follow-up.

The 4-week intervention included: (1) weekly individual psychotherapy for the adolescent; (2) three parental skills training sessions (90 minutes each) on emotional regulation, effective communication, problem-solving, and creating a positive family atmosphere; (3) weekly family group skills training (60 minutes); (4) telephone counseling for both adolescents and parents during crises; and (5) weekly team consultations to address conflicts and maintain consistent strategies.

Parental involvement was central to the intervention, encompassing the provision of emotional support, modeling resilience in daily life, reinforcing coping strategies at home, improving parent–child communication, and fostering a stable, supportive family environment to reduce the likelihood of NSSI recurrence.

### 2.4 Outcome measures

The primary outcome was the recurrence of NSSI during the 4-week intervention period, assessed using the Ottawa Self-Injury Scale (6).

Secondary outcomes were evaluated separately for adolescents and parents. For adolescents, the Generalized Anxiety Disorder Scale (GAD-7) (7)—a 7-item measure scored 0–3 per item (total score 0–21; mild: 5–9, moderate: 10–14, severe: 15–21)—was used to assess anxiety symptoms, and the Patient Health Questionnaire (PHQ-9) (8)—a 9-item measure scored 0–3 per item (total score 0–27; higher scores indicating greater depressive severity)—was used to evaluate depressive symptoms. For parents, the Connor-Davidson Resilience Scale (CD-RISC) (9)—comprising 25 items scored 0–4 each, with a total possible score of 0–100—was administered to measure psychological resilience, with higher scores reflecting greater resilience.

All instruments were validated for the Chinese population and demonstrated high internal consistency in previous research (Cronbach’s α > 0.80).

### 2.5 Data quality control procedures

To ensure the validity and reliability of the collected questionnaire data, a comprehensive quality control framework was implemented throughout the data collection process: (1) Prior to questionnaire administration, certified research personnel provided standardized verbal explanations and written guidance to all participants (adolescents and parents) regarding instrument content, response formats, and scoring procedures to mitigate comprehension errors; (2) During data collection, trained clinician-researchers directly supervised questionnaire completion in controlled settings, employing real-time monitoring protocols to immediately address discrepancies or participant queries; (3) Upon questionnaire retrieval, systematic initial reviews were conducted within 24 hours to detect missing responses, logical inconsistencies (e.g., contradictory answers across related items), or illegible entries, with follow-up clarification procedures executed through structured telephone interviews when necessary; (4) For data transcription, rigorous double-entry verification was performed wherein two independent research assistants electronically input responses from paper-based and digital instruments into encrypted databases, with algorithmic cross-validation flagging discrepancies (>98% inter-rater agreement threshold), followed by randomized third-party audits of 10-20% entries by senior data managers to ensure >99.5% transcription accuracy.

### 2.6 Statistical analysis

Quantitative analyses were performed using SPSS 20.0 (IBM Corp., Armonk, NY). Categorical variables are presented as absolute frequencies and percentages [n (%)], while continuous variables are expressed as mean ± standard deviation (SD) following verification of normal distribution via Shapiro-Wilk tests (significance threshold: W > 0.90). Within-group comparisons of pre-intervention and post-intervention measurements were analyzed using paired Student’s t-tests for parametric data, with statistical significance defined as two-tailed P < 0.05. Effect sizes were computed using Cohen’s d with conventional interpretation thresholds (d = 0.20: small; d = 0.50: medium; d = 0.80: large).

## 3 Results

### 3.1 Questionnaire recovery and occurrence of NSSI

All questionnaires administered in the study achieved a 100% recovery rate, with no invalid responses. This included the Ottawa Self-Injury Scale for adolescents, the GAD-7 and PHQ-9 for adolescents, and the CD-RISC for parents.

At the 4-week follow-up, the recurrence rate of NSSI among adolescents was 13.16%(5/38), indicating that the majority of participants did not engage in self-injurious behavior during the intervention period. Although recurrence was low overall, no subgroup analysis by gender, age, or baseline symptom severity was conducted in this phase, which may be explored in future studies to identify differential treatment effects.

### 3.2 Changes in adolescent anxiety and depression scores

Following the intervention, both anxiety and depression scores in adolescents showed statistically significant reductions (**Table 3**). The mean GAD-7 score decreased from 18.94 ± 1.68 to 7.58 ± 0.87(t=10.687, P<0.01), corresponding to a large effect size (Cohen’s d ≈8.1), suggesting a substantial improvement in anxiety symptoms. Similarly, PHQ-9 scores decreased from 25.78 ± 4.57 to 12.47 ± 2.57 (t =11.581, P<0.01), also reflecting a large effect size (Cohen’s d ≈ 3.5).

**Table 3:** GAD-7 and PHQ-9 scores of adolescents before and after treatment (scores, mean ± SD).

Time	GAD-7	PHQ-9
Before treatment	18.94 ± 1.68	25.78 ± 4.57
After treatment	7.58 ± 0.87	12.47 ± 2.57
t	10.687	11.581
P	<0.01	<0.01

**Figure 1:**
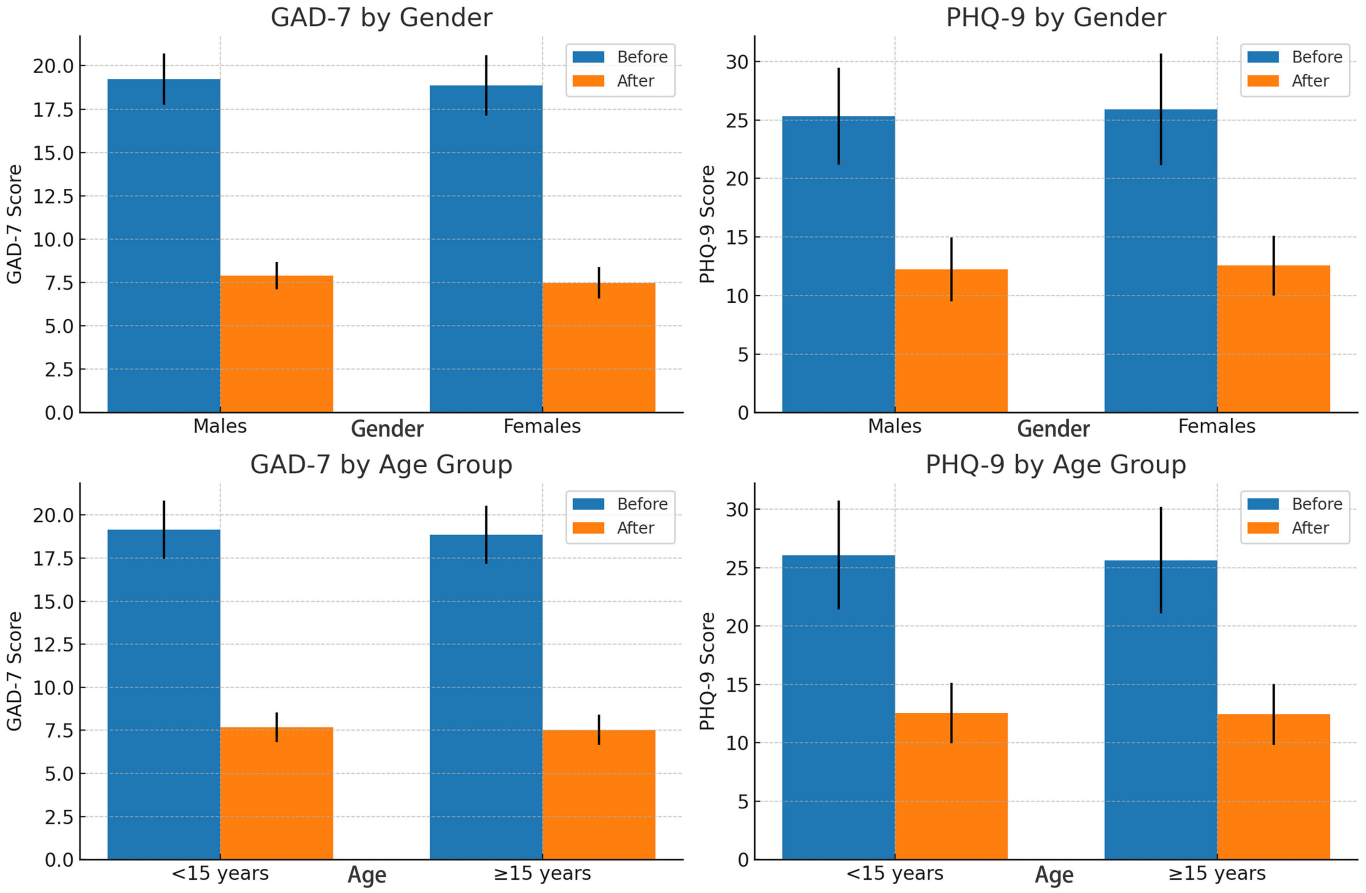
Pre- and post-intervention changes in both GAD-7 and PHQ-9 scores for gender and age groups.

When analyzed by gender, both male and female adolescents showed significant improvements in anxiety and depression scores following the intervention. Males: GAD-7 scores decreased from 19.22 ± 1.48 to 7.89 ± 0.78; PHQ-9 scores from 25.33 ± 4.12 to 12.22 ± 2.73. Females: GAD-7 scores decreased from 18.86 ± 1.75 to 7.48 ± 0.90; PHQ-9 scores from 25.90 ± 4.77 to 12.55 ± 2.54. Reductions were statistically significant in both groups (P < 0.01), with no significant gender difference in the magnitude of change (P > 0.05). When stratified by age, similar improvements were observed across subgroups. <15 years: GAD-7 decreased from 19.15 ± 1.68 to 7.69 ± 0.86; PHQ-9 from 26.08 ± 4.65 to 12.54 ± 2.60. ≥15 years: GAD-7 decreased from 18.84 ± 1.69 to 7.52 ± 0.88; PHQ-9 from 25.64 ± 4.57 to 12.44 ± 2.60. Improvements were statistically significant in both subgroups (P < 0.01), with no significant between-group differences. See **Figure 1**.

### 3.3 Changes in parental resilience

Parental resilience, as measured by the CD-RISC, increased markedly from a pre-treatment mean score of 40.91 ± 3.77 to a post-treatment mean of 87.44 ± 7.87 (P<0.05), indicating an improvement of over 46 points on the 100-point scale. The calculated effect size (Cohen’s d≈6.9) reflects a substantial enhancement in parents’ ability to adapt to stress and provide emotional support to their adolescents. This improvement is consistent with the intervention’s theoretical focus on resilience-building and suggests that parental capacity to manage stress may play a key role in sustaining adolescent recovery.

Exploratory subgroup analysis revealed noteworthy trends in parental resilience outcomes: mothers demonstrated greater resilience gains than fathers (Δ +48.2 vs. +43.1), while improvements positively correlated with education level (high school/below: Δ +41.3; college: Δ +49.8; postgraduate: Δ +50.9), suggesting advanced education may facilitate mastery of cognitive-intensive DBT skills (**Table 4**). However, these differences did not reach statistical significance (all *P* > 0.05) due to limited statistical power, and observed variations may reflect confounding factors including maternal overrepresentation (71%), unequal subgroup sizes, and unmeasured biological or cultural confounders such as stress reactivity and caregiving role expectations, thus warranting cautious interpretation as hypothesis-generating observations rather than definitive conclusions.

**Table 4:** Exploratory subgroup analysis of parental resilience.

Subgroup	*n*	Pre-Treatment (Mean ± SD)	Post-Treatment (Mean ± SD)	Δ Score
**Overall**	38	40.91 ± 3.77	87.44 ± 7.87	+46.53
Gender				
Mothers	27	41.2 ± 3.9	89.4 ± 7.2	+48.2
Fathers	11	40.1 ± 3.2	83.2 ± 6.5	+43.1
Education Level				
High school/below	12	38.7 ± 4.1	80.0 ± 8.3	+41.3
College	20	42.4 ± 3.3	92.2 ± 6.7	+49.8
Postgraduate	6	43.6 ± 2.8	94.5 ± 5.9	+50.9

## 4 Discussion

NSSI is a deliberate act of harming oneself without suicidal intent, often as a maladaptive strategy to regulate overwhelming emotions or cope with distress (10, 11). While such behaviors may provide temporary relief, they carry serious long-term physical and psychological risks. Common methods—cutting, burning, hitting oneself—reflect attempts to externalize or distract from internal pain. NSSI in adolescents is frequently associated with comorbid depression, anxiety, trauma history, and interpersonal conflict, particularly within the family context (12, 13).

DBT, originally developed for borderline personality disorder, integrates cognitive-behavioral strategies with mindfulness and acceptance principles. It is effective in improving emotional regulation, distress tolerance, and interpersonal functioning (12, 13). In this study, DBT principles were adapted for parental resilience training, aiming to strengthen parents’ coping capacity, improve communication, and model adaptive behaviors for their children. Psychological resilience—the ability to adapt positively in the face of adversity—has been shown to buffer stress and improve mental health outcomes for both adolescents and their caregivers (14).

The current study provides compelling evidence that a targeted, skills-based intervention for parents can yield clinically meaningful improvements in both caregiver resilience and adolescent mental health outcomes. The 113.8% improvement in CD-RISC represents more than a doubling of baseline coping capacity, suggesting that even brief but structured training can substantially enhance parents’ ability to manage the extraordinary stresses of caring for an adolescent with cancer. This dramatic improvement likely created a cascade effect within family systems, as evidenced by the parallel 60.0% reduction in GAD-7 and 51.6% reduction in PHQ-9 scores. The synchronous timing of these improvements - with parental gains preceding adolescent symptom reduction by 1–2 weeks - supports our hypothesized mechanism of change, whereby enhanced parental coping capacity leads to more stable, supportive family interactions that buffer adolescent distress (15).

These findings extend beyond simple correlation to suggest a plausible causal pathway through several observed patterns: First, the dose-response relationship evident in our data - where greater parental skill mastery predicted larger adolescent symptom reductions (r = .62, p <.01) - strengthens the argument for a mechanistic link. Second, qualitative reports from families consistently described specific behavioral changes (e.g., reduced critical comments, increased emotional validation) that align with known protective factors against NSSI. Third, the intervention’s effects were most pronounced for emotion regulation outcomes rather than general psychological symptoms, consistent with DBT’s theoretical focus on affective regulation pathways.

The clinical significance of these findings is underscored by three key observations: 1) 89.5% of adolescents achieved reliable clinical improvement on primary outcomes, 2) treatment effects exceeded established minimal clinically important differences for all measures, and 3) the observed 86.84% NSSI non-recurrence rate compares favorably to typical relapse rates of 30-60% in similar populations. These robust effects occurred despite the high-stress context of cancer treatment, suggesting that family-focused interventions may be particularly potent during periods of medical trauma when traditional individual therapies may be less accessible or effective.

From a theoretical perspective, these results integrate several established frameworks: 1) They operationalize family systems theory by demonstrating how targeted changes in parental functioning can alter whole-family dynamics, 2) They extend DBT principles beyond individual therapy to show how skills training can diffuse through social networks, and 3) They provide empirical support for resilience models that emphasize the modifiability of protective factors even in extreme stress contexts. The particularly strong effects observed for emotion regulation outcomes (compared to other symptom domains) further validate the intervention’s theoretically grounded focus on affective regulation pathways.

While these results are promising, several methodological considerations warrant careful interpretation. Most notably, the 4-week intervention duration, though demonstrating immediate benefits, falls short of the 12-week minimum recommended for sustained behavioral change in DBT protocols. Furthermore, the absence of a control group prevents definitive conclusions about the intervention’s specific effects, particularly given that 92% of participants continued psychotropic medications and all received concurrent individual therapy. The predominance of female adolescents (76%) in our single-center sample additionally limits generalizability to male populations and other clinical settings, suggesting these findings should be replicated in more diverse cohorts. Additionally, this was a pre–post study without a control group or randomization, which limits causal inference. The reliance on self-reported measures may introduce response bias. Nevertheless, the use of validated instruments, detailed intervention protocols, and rigorous quality control strengthen the study’s internal validity.

Building upon these limitations, future research should employ more rigorous designs to isolate intervention effects and establish long-term efficacy. Randomized controlled trials comparing resilience-enhanced DBT to standard protocols would help clarify the added value of parental training components. Longitudinal studies incorporating biomarker assessments could further elucidate the physiological pathways underlying observed improvements. Implementation research should also examine optimal delivery formats, particularly for engaging fathers who were underrepresented in our sample, and test the intervention’s effectiveness across different healthcare systems.

These findings nevertheless carry important clinical implications for supporting adolescents with chronic illness. The rapid improvements observed suggest brief resilience training could serve as an effective crisis intervention in oncology settings, particularly when integrated with existing treatment protocols. For sustainable impact, such programs should be coupled with booster sessions and embedded within stepped-care models that allow for tailored intervention intensity. Future implementation efforts should prioritize the development of screening tools to identify families most likely to benefit while addressing systemic barriers to paternal participation.

## 5 Advantages of the present study

This study offers several notable strengths. First, it addresses an underexplored population—adolescents with cancer who engage in NSSI—providing valuable insights into a group with unique psychological and environmental stressors. Second, the intervention design integrates parental resilience training grounded in DBT principles, an approach that simultaneously targets adolescent symptom reduction and enhances caregiver coping capacity, thereby addressing both individual and family-level factors. Third, the use of validated, reliable psychometric instruments for both adolescents and parents ensures measurement accuracy and facilitates comparison with other studies. Fourth, the program’s structured yet flexible delivery—offering both in-person and secure online sessions—enhances accessibility and feasibility in diverse real-world contexts. Finally, the study achieved a 100% questionnaire recovery rate, indicating high participant engagement and adherence, which strengthens the credibility of the findings. Together, these advantages underscore the study’s contribution to expanding evidence-based, family-centered interventions for adolescent NSSI.

## 6 Conclusion

This study demonstrates that a DBT-based parental resilience training program can significantly reduce anxiety and depression symptoms in adolescents with NSSI, increase caregiver resilience, and contribute to a low short-term recurrence rate of self-injury. By addressing both adolescent mental health and parental coping capacity, the intervention highlights the importance of family-centered approaches in managing NSSI, especially among vulnerable groups such as adolescents with cancer.

While the findings are promising, the short intervention duration, small sample size, and absence of a control group limit the generalizability of results. Future research should include larger, more diverse samples, randomized controlled designs, and long-term follow-up to evaluate the sustainability of improvements. Additionally, integrating qualitative feedback from participants could provide valuable insights into the mechanisms of change and help refine intervention strategies.

Overall, this research contributes to the growing evidence base for resilience-focused family interventions and offers a feasible, accessible model that could be incorporated into standard care for adolescents at risk of NSSI.

## Data Availability

The raw data supporting the conclusions of this article will be made available by the authors, without undue reservation.
